# Supplementary material for: TT-Mars: structural variants assessment based on haplotype-resolved assemblies
Source: Genome Biol. 2022 May 6;23:110. doi: 10.1186/s13059-022-02666-2 (PMC9077962; doi:10.1186/s13059-022-02666-2)
Supplement: Supplementary file 1 — Additional file 1 Supplementary figures and analysis. [file 13059_2022_2666_MOESM1_ESM.pdf]

Additional file 1

1 Analysis of TT-Mars additionally analyzed calls

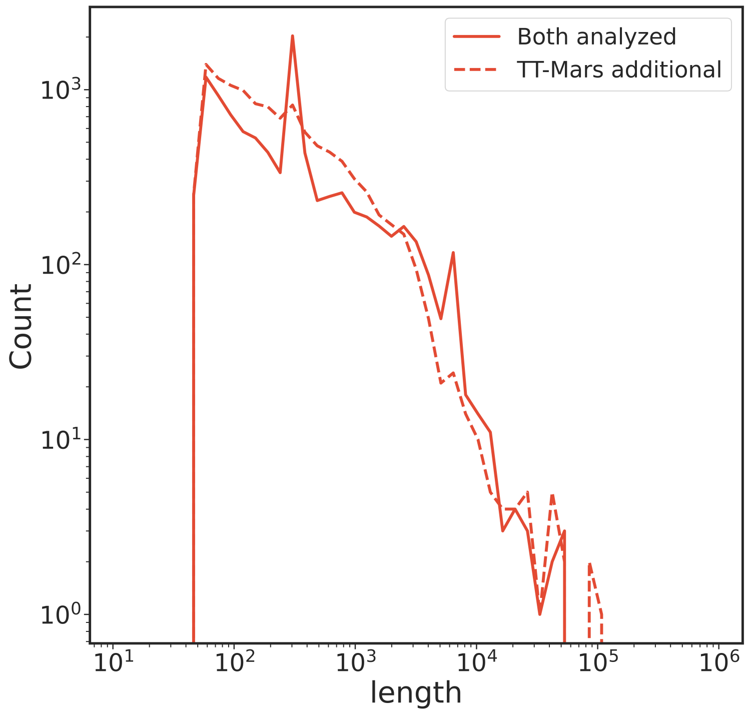

Fig. S1: Length distribution of calls that analyzed by both TT-Mars and truvari and analyzed by TT-Mars only on pbsv HG002 callset.

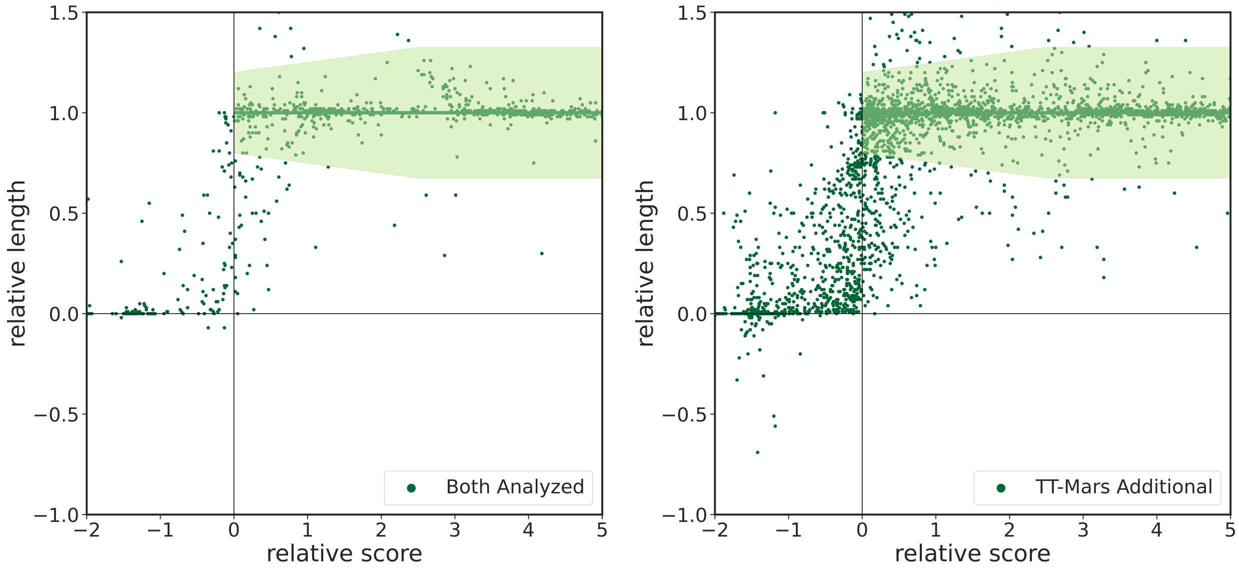

Fig. S2: Scatter plot of TT-Mars scores of calls that analyzed by both TT-Mars and truvari (left) and analyzed by TT-Mars only (right) on pbsv HG002 callset.

2 Fraction of excluded genome

Table S1: Fraction of genome that is excluded by TT-Mars. Including regions that are not covered by 1 or 2 haplotypes of the assembly and the centromere.

Genome	HG00096	HG00171	HG00513	HG00731	HG00732
Fraction	9.9%	10.0%	9.3%	9.5%	9.3%
Genome	HG00864	HG01596	HG03009	HG01114	HG01505
Fraction	9.7%	10.1%	10.0%	10.2%	9.9%

3 Analysis of variability of TP rate

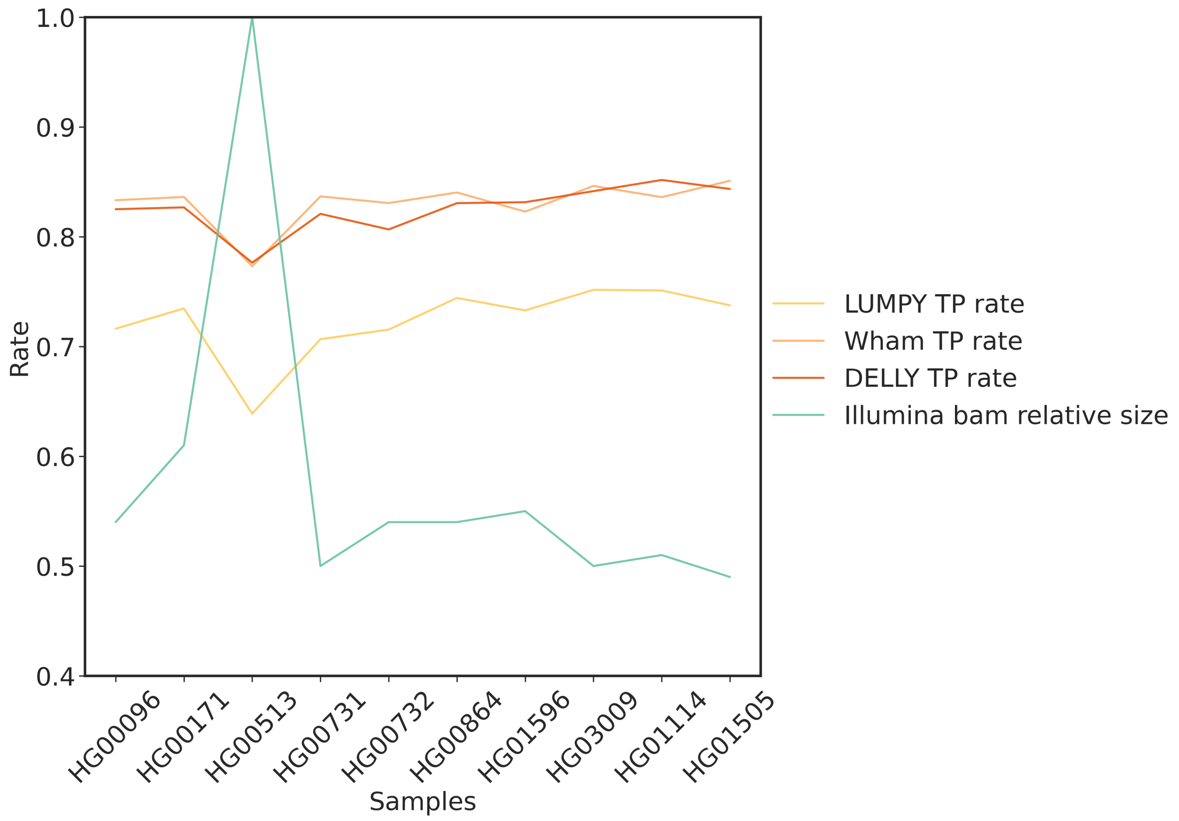

Fig. S3: BAM size and TP rate among short read algorithms.

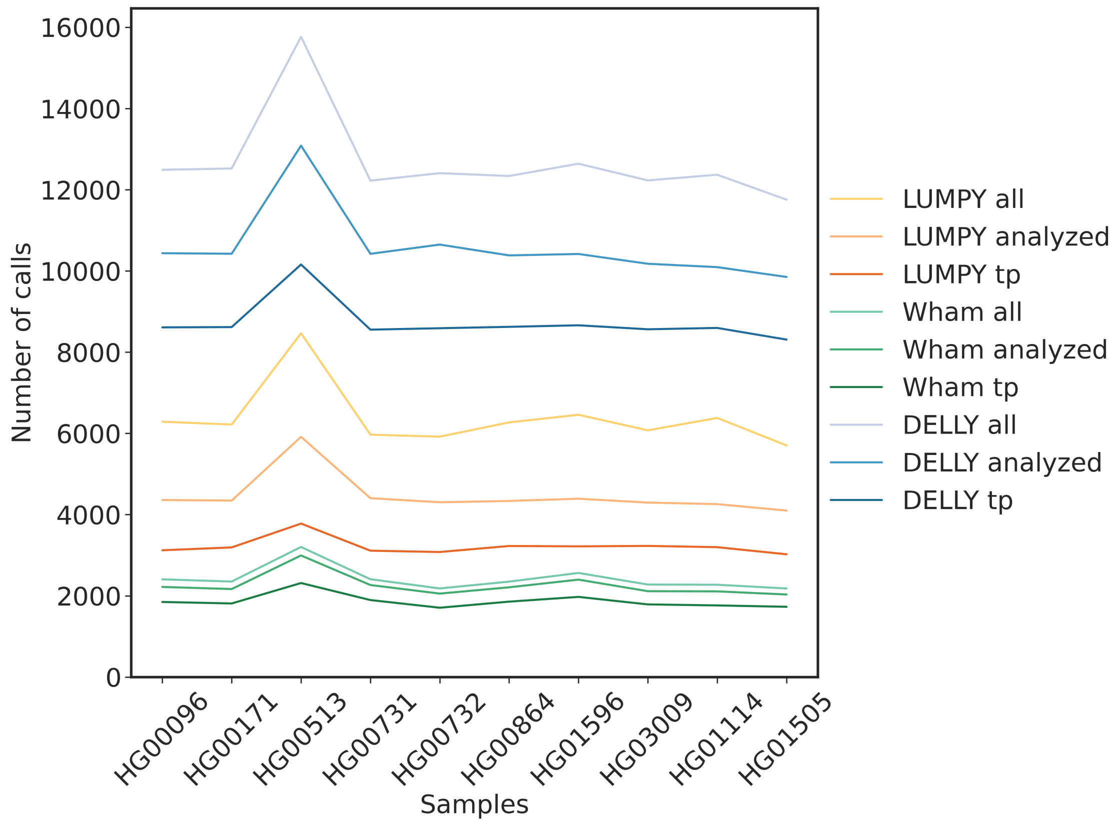

Fig. S4: The number of all the calls, analyzed calls and TP calls per genome.

4 Dipcall+truvari and TT-Mars classification results loci

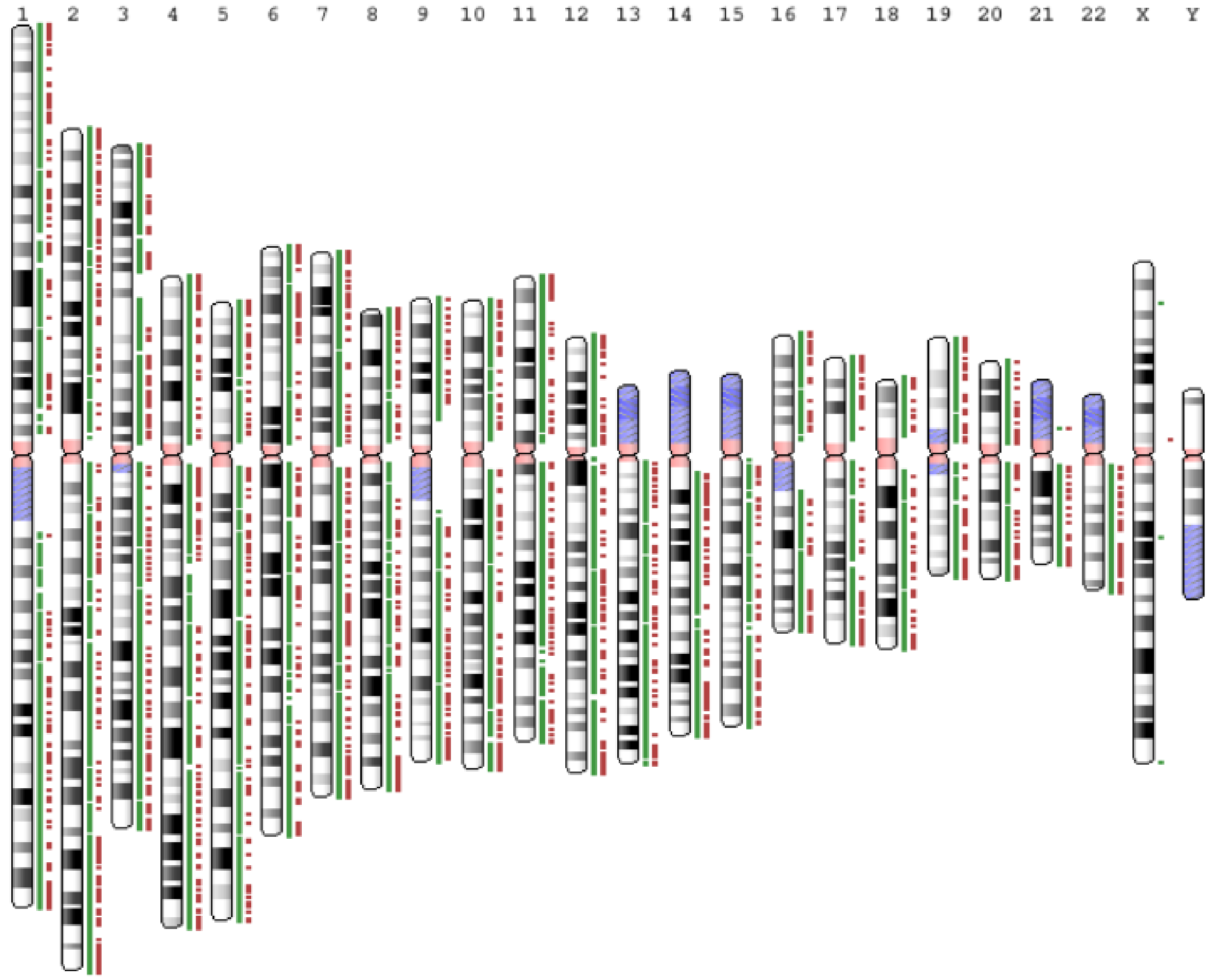

Fig. S5: Loci of dipcall+truvari and TT-Mars calls from the HG00096 pbsv call set. Green bars are the calls that two method agree on, red bars are the calls that two method disagree on.

5 Curation of dipcall+truvari and TT-Mars SV annotation

On the pbsv call set of sample HG00096, we randomly selected 100 calls that are given different results by dipcall+truvari and TT-Mars (50 calls dipcall+truvari FP TT-Mars TP and 50 calls dipcall+truvari TP TT-Mars FP) for manual inspection in IGV. In each figure, the first track is the pbsv call set, the second track is the dipcall call set. The following two tracks are alignments used to generate the orthology map in TT-Mars (lra), the next two tracks are alignments used by dipcall (pbmm2). The track at bottom is the tandem duplication regions. Annotation of each figure is in the caption.

5.1 Dipcall+Truvari FP and TT-Mars TP results

Category I: a call made by pbsv is fragmented into multiple calls by dipcall. Category II: there are correct variants near by and the validation depends on the genomic region considered by truvari. Category III: No matched SV in the alignments generated by minimap2 that are used for validation, but do show matched SV(s) in the LRA based alignments.

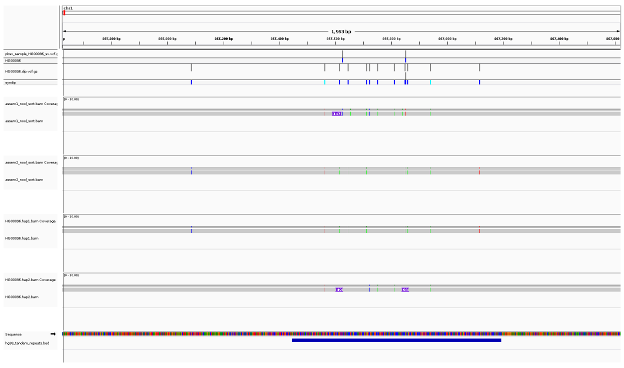

Fig. S6: Category I

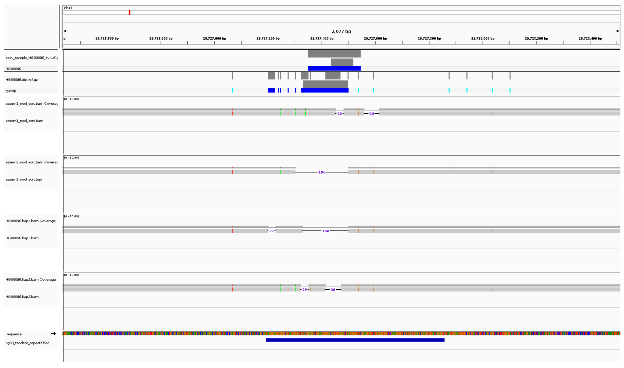

Fig. S7: Category I

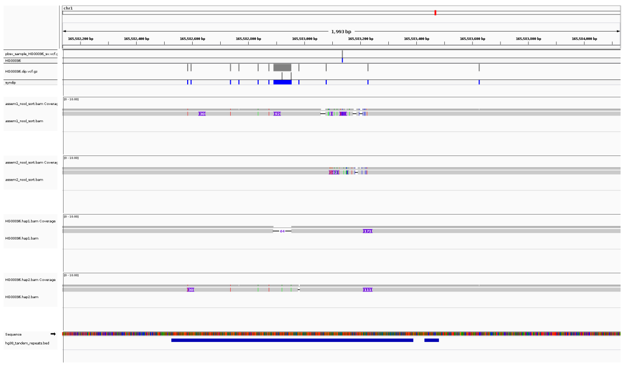

Fig. S8: Category I

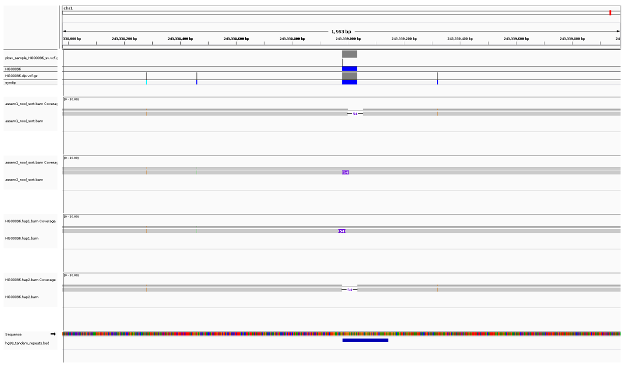

Fig. S9: Category II

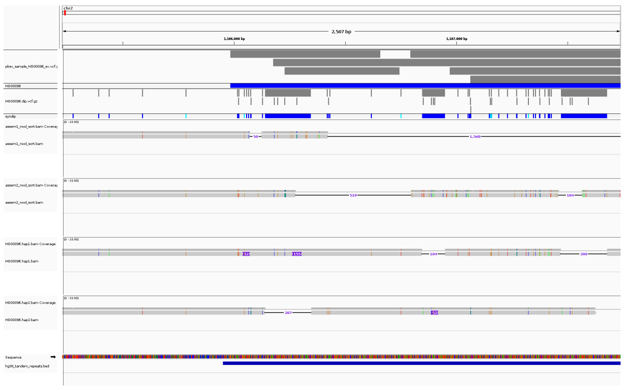

Fig. S10: Category I

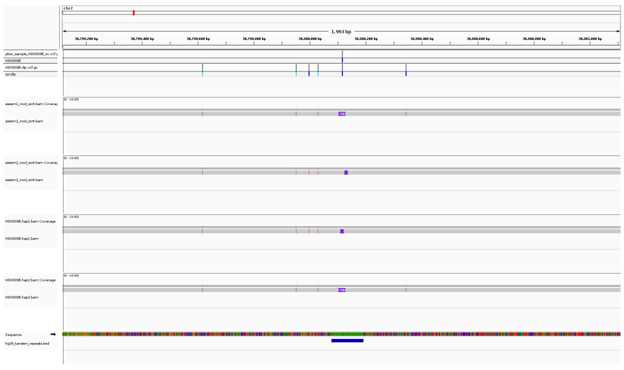

Fig. S11: Category II

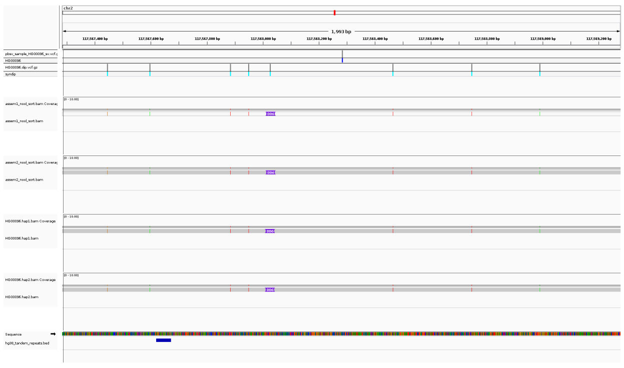

Fig. S12: Category II

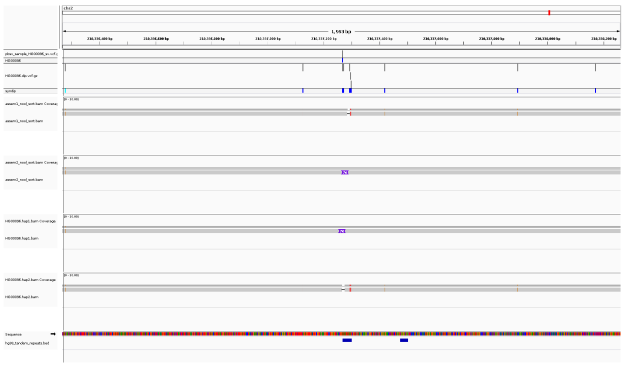

Fig. S13: Category II

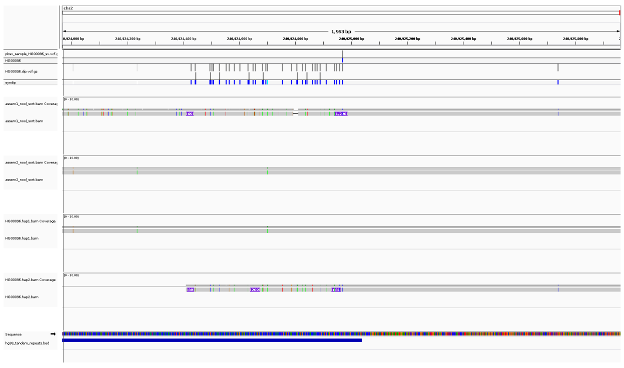

Fig. S14: Category III

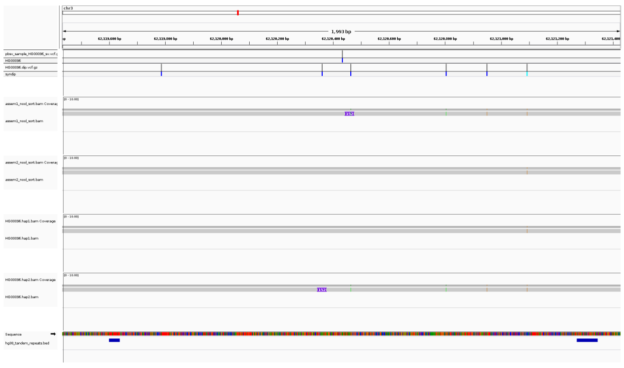

Fig. S15: Category II

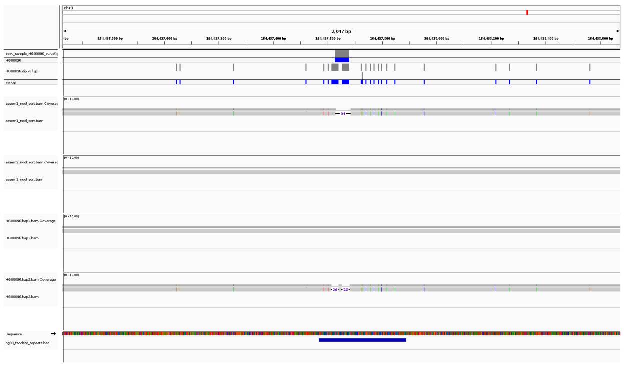

Fig. S16: Category I

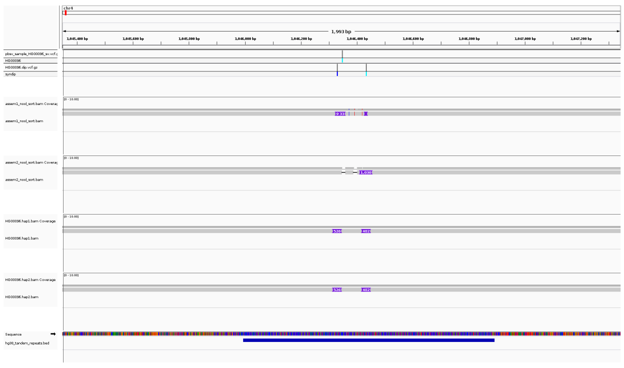

Fig. S17: Category I

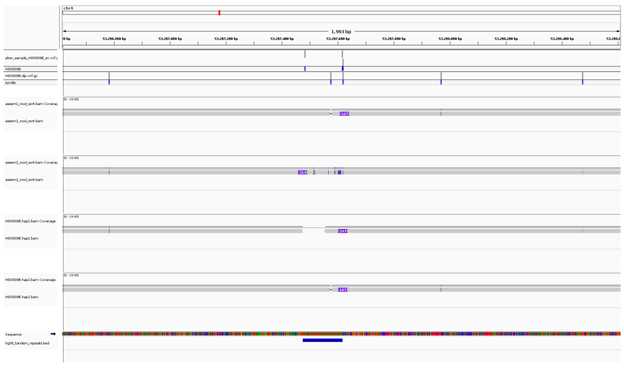

Fig. S18: Category III

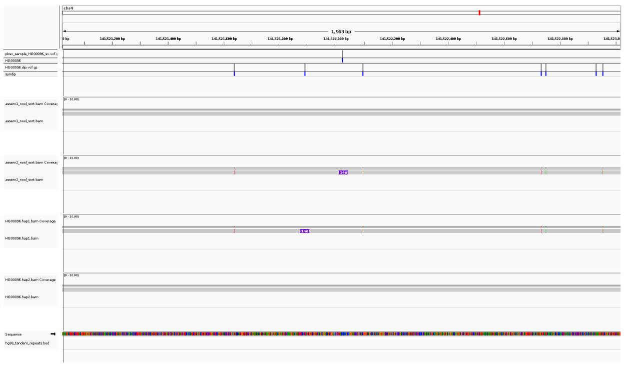

Fig. S19: Category II

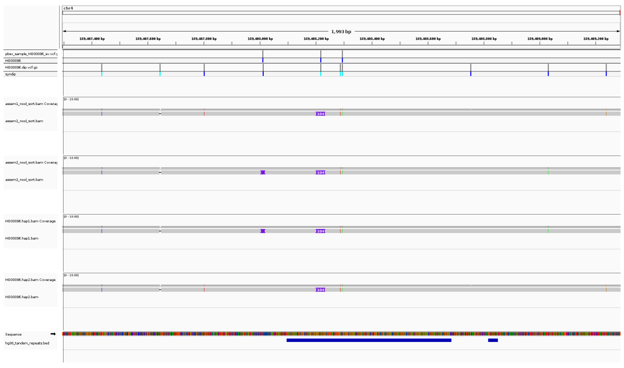

Fig. S20: Category II

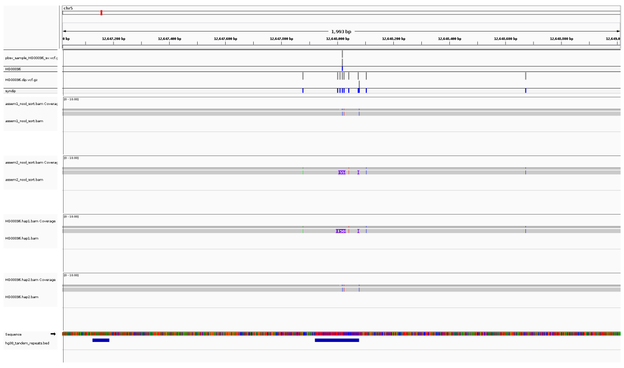

Fig. S21: Category I

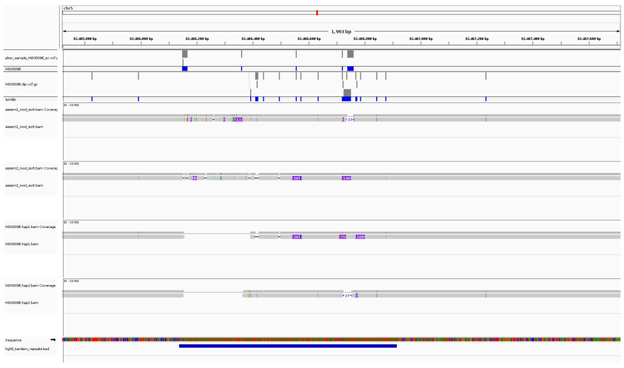

Fig. S22: Category I

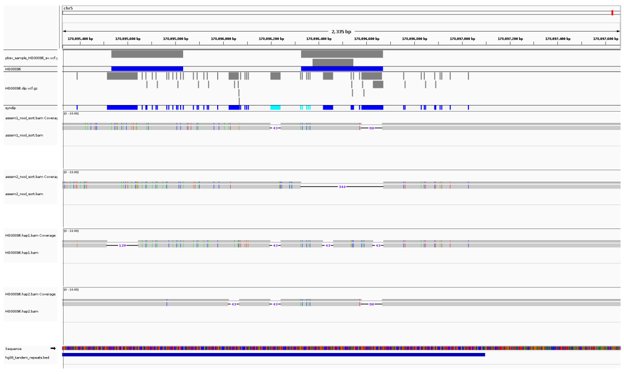

Fig. S23: Category III

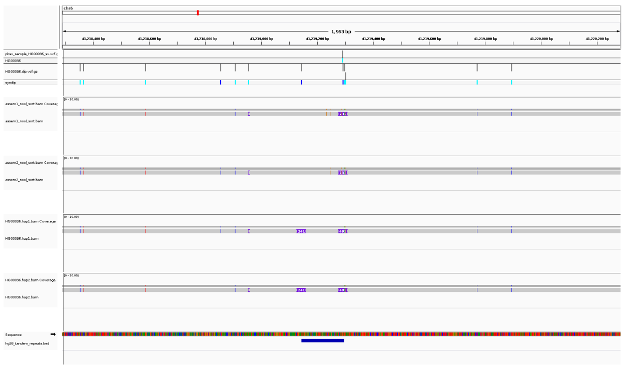

Fig. S24: Category I

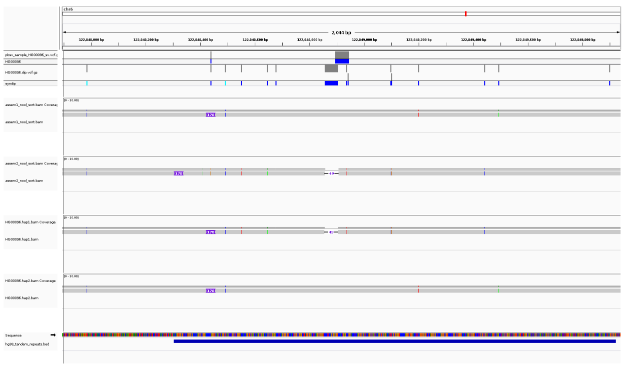

Fig. S25: Category II

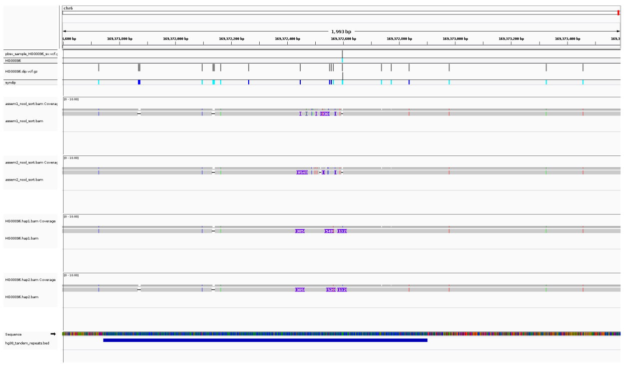

Fig. S26: Category I

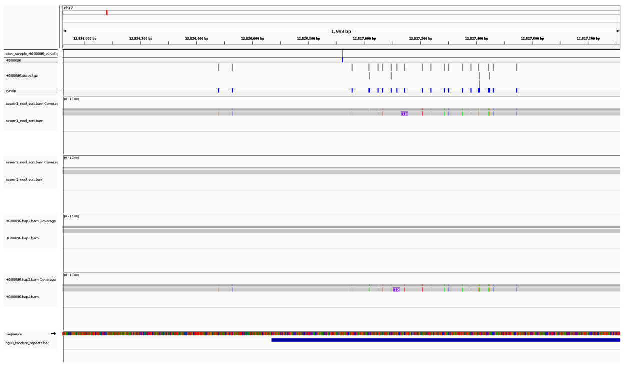

Fig. S27: Category II

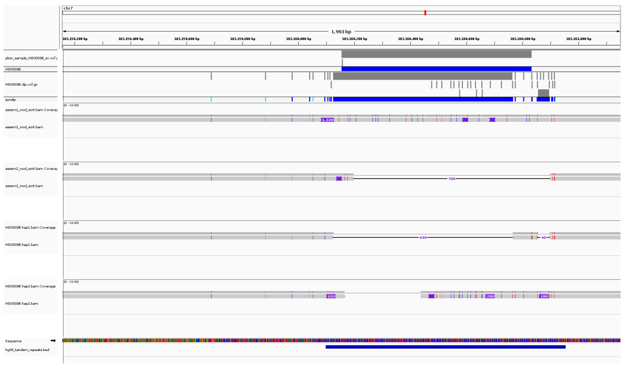

Fig. S28: Category I

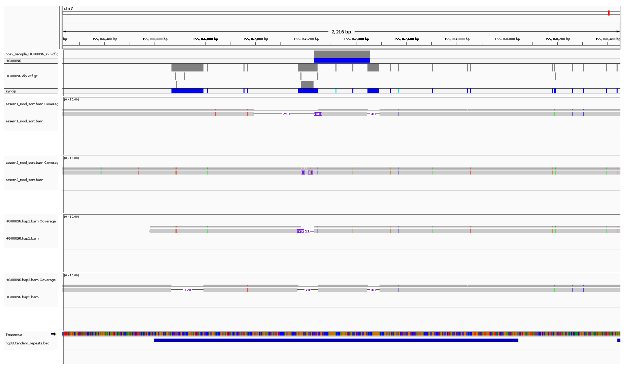

Fig. S29: Category I

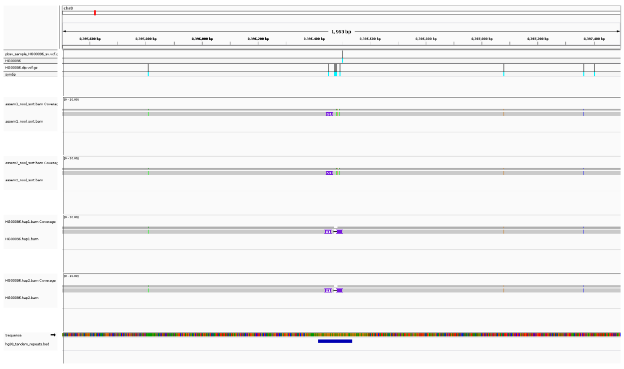

Fig. S30: Category I

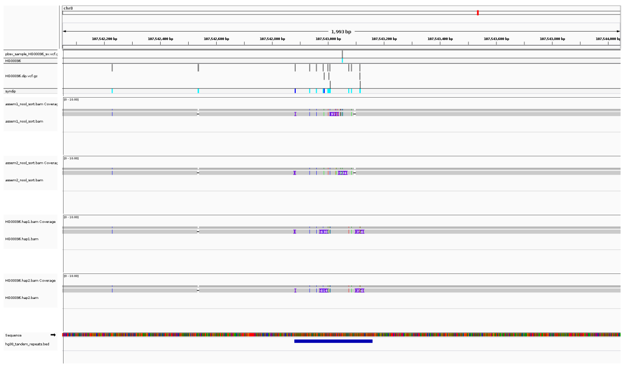

Fig. S31: Category I

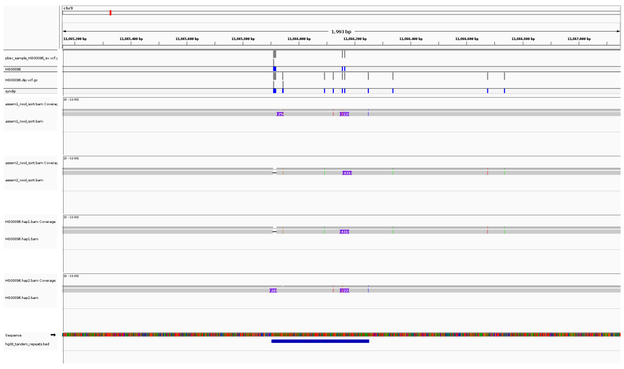

Fig. S32: Category II

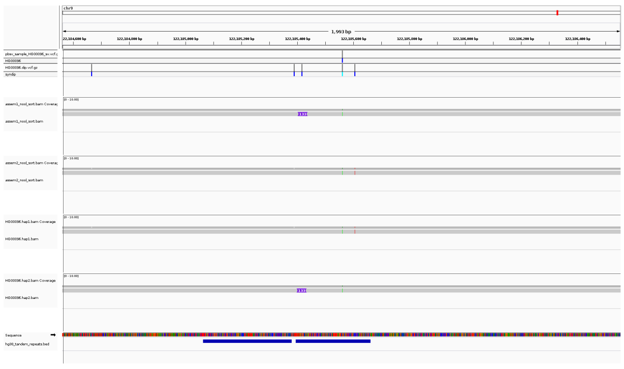

Fig. S33: Category II

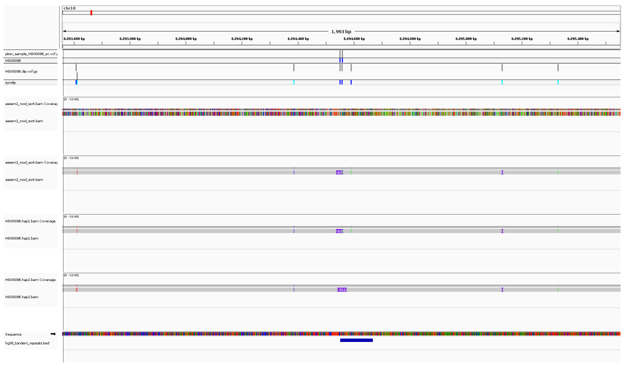

Fig. S34: Category II

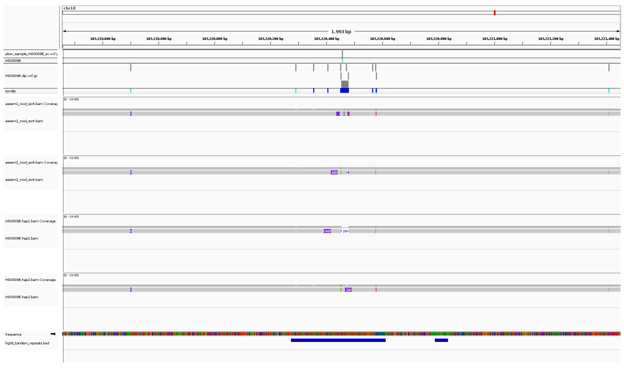

Fig. S35: Category I

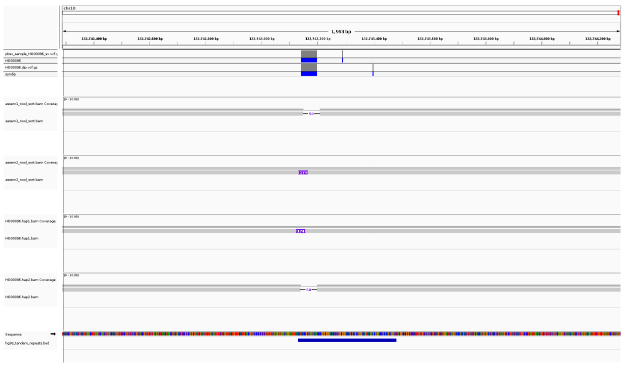

Fig. S36: Category II

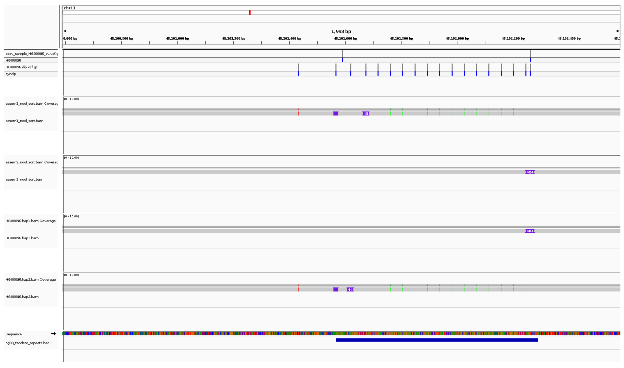

Fig. S37: Category I

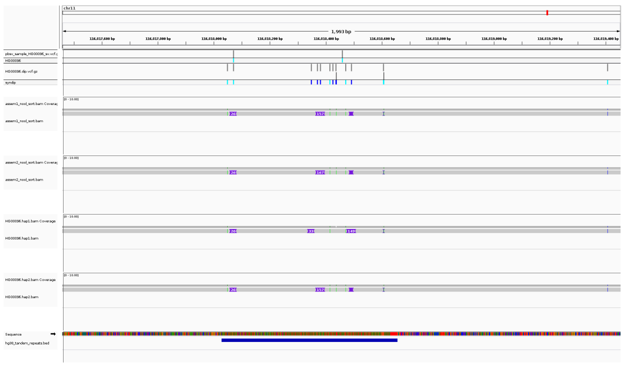

Fig. S38: Category I

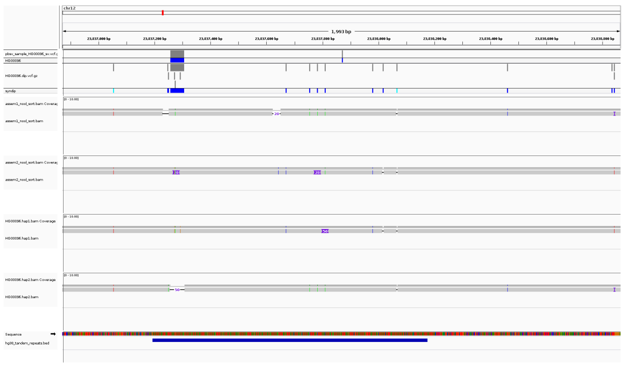

Fig. S39: Category II

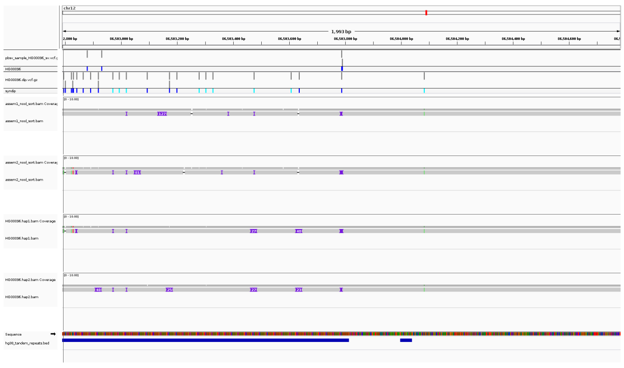

Fig. S40: Category I

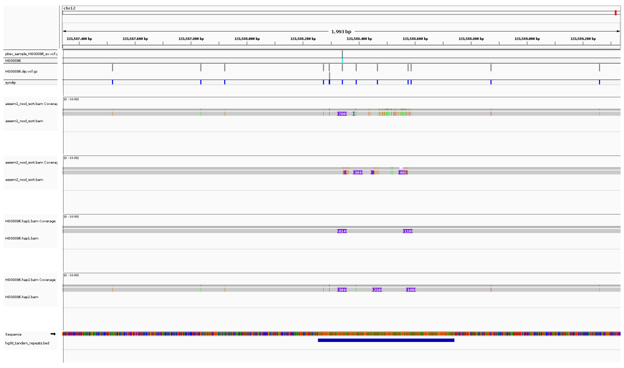

Fig. S41: Category I

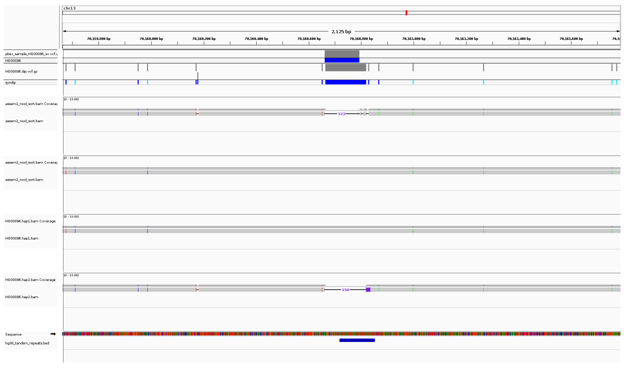

Fig. S42: Category I

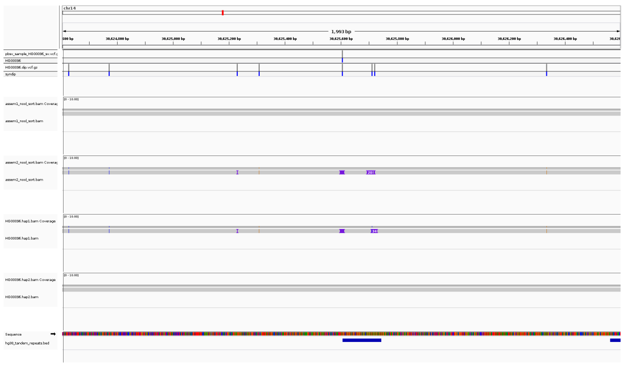

Fig. S43: Category I

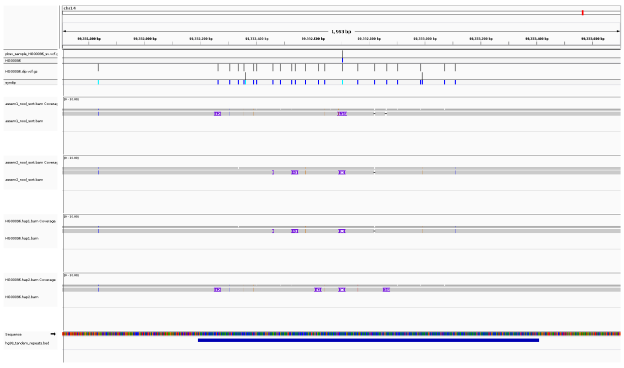

Fig. S44: Category I

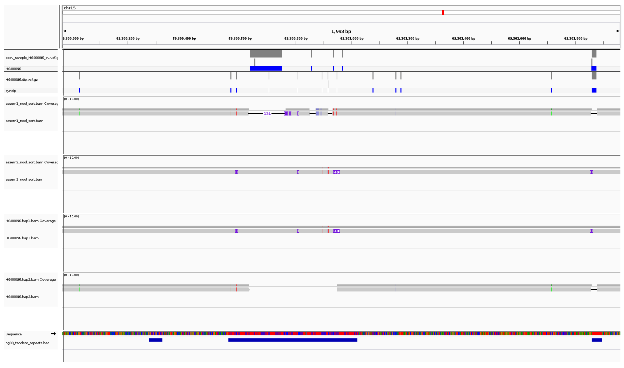

Fig. S45: Category III

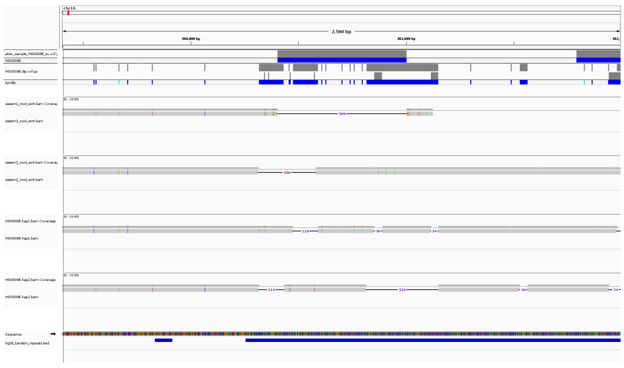

Fig. S46: Category I

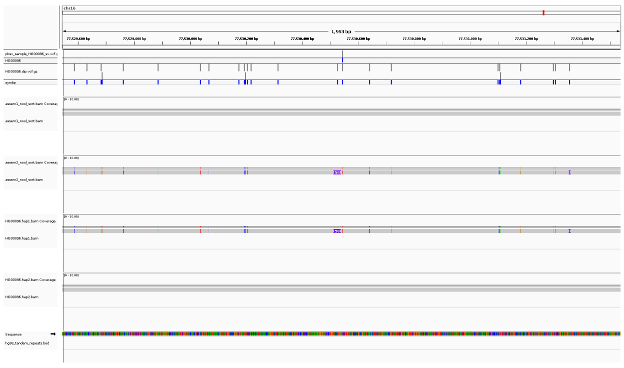

Fig. S47: Category II

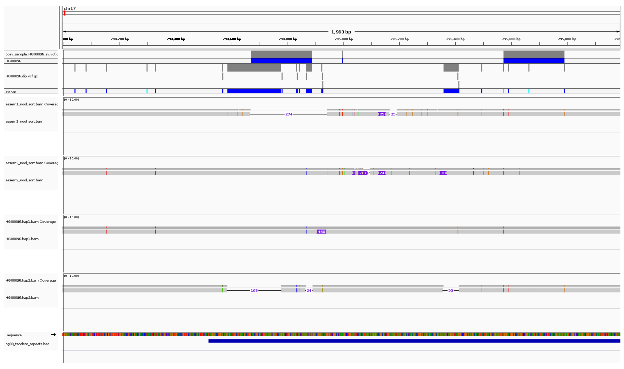

Fig. S48: Category I

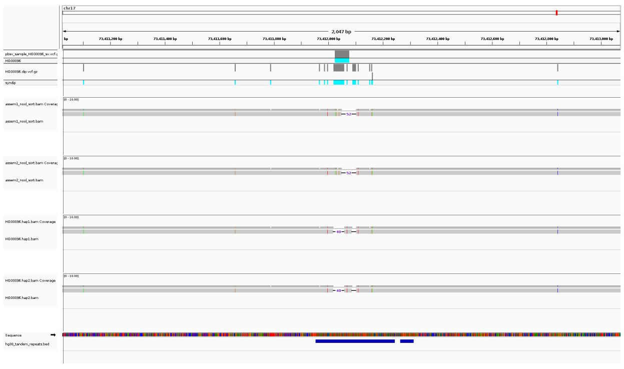

Fig. S49: Category I

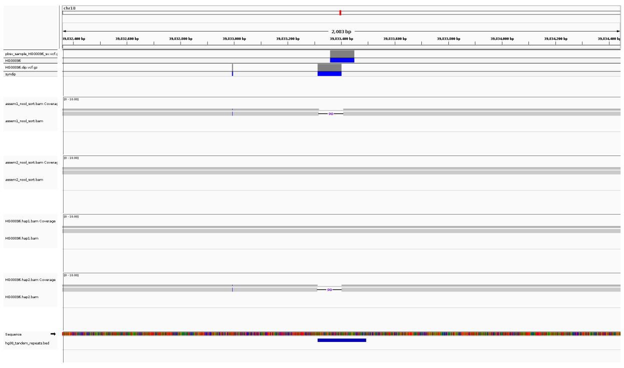

Fig. S50: Category II

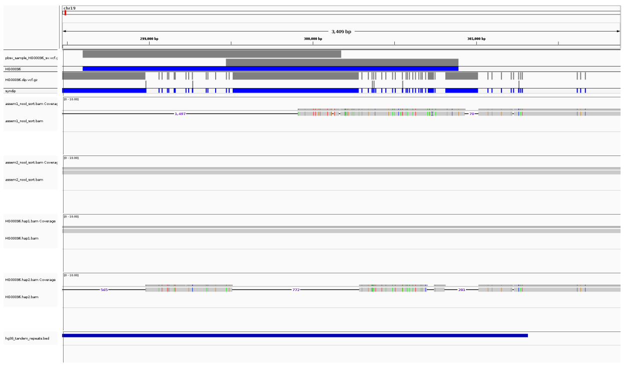

Fig. S51: Category I

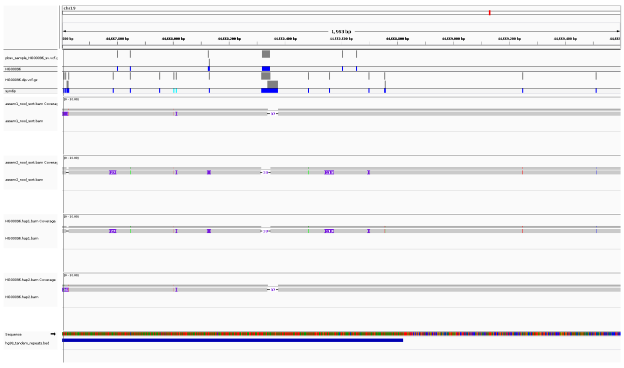

Fig. S52: Category II

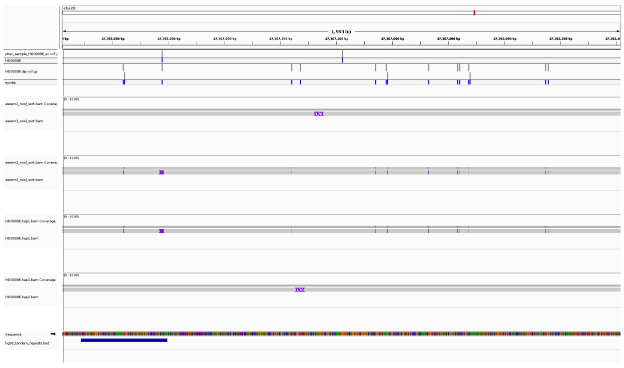

Fig. S53: Category II

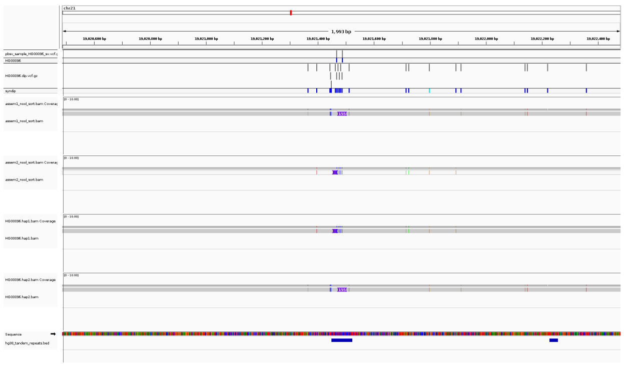

Fig. S54: Category II

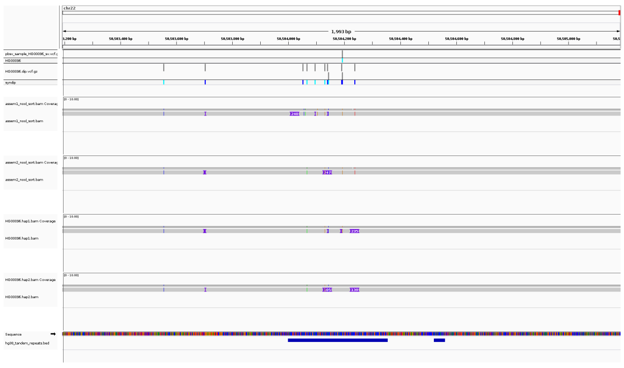

Fig. S55: Category I

5.2 Dipcall+Truvari TP and TT-Mars FP results

Category I: no matched SV in the assembly alignment used by dipcall. Category II: exist matched SVs in the assembly used by TT-Mars. Category III: others (not category I and not category II).

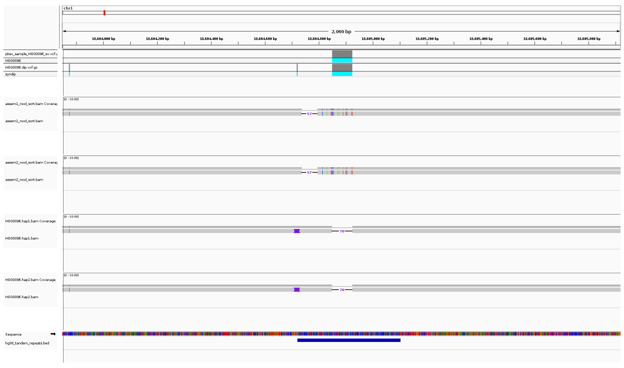

Fig. S56: Category III

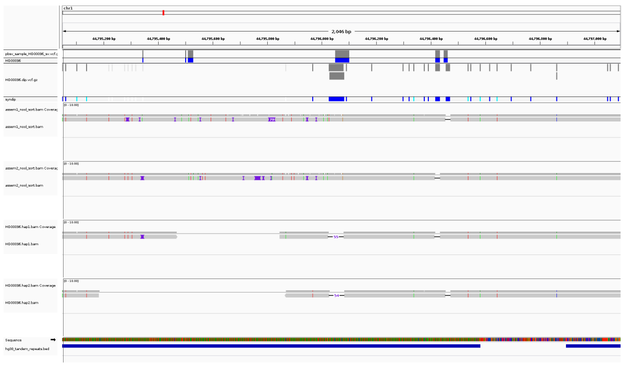

Fig. S57: Category III

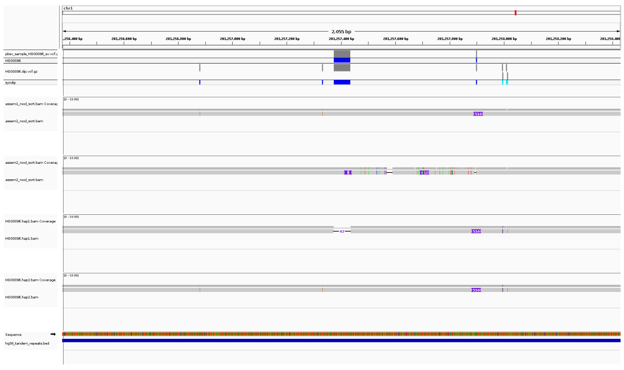

Fig. S58: Category III

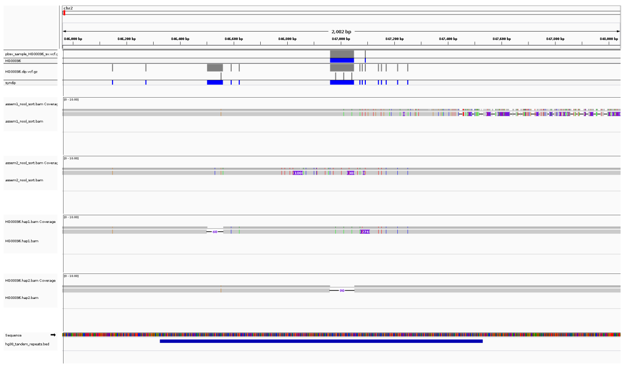

Fig. S59: Category III

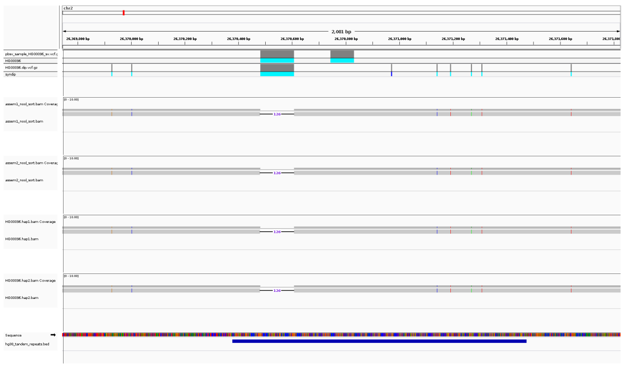

Fig. S60: Category III

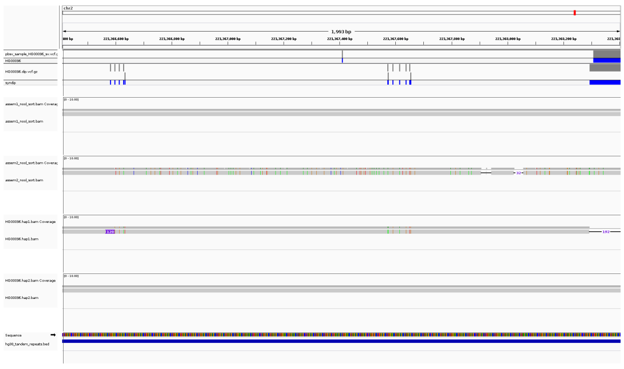

Fig. S61: Category III

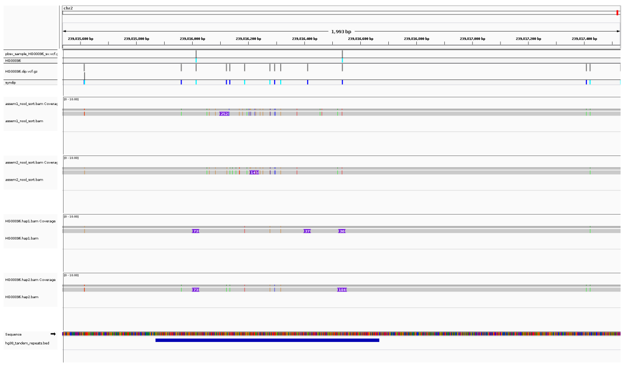

Fig. S62: Category III

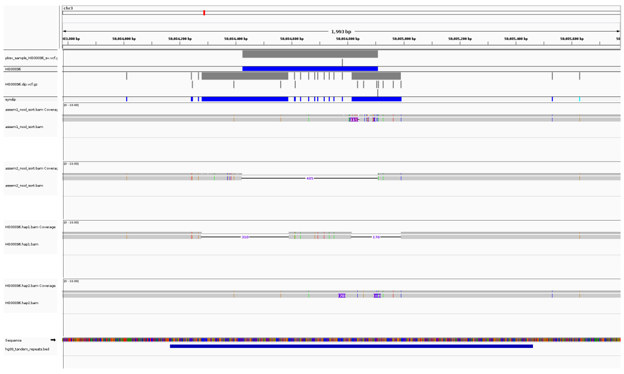

Fig. S63: Category III

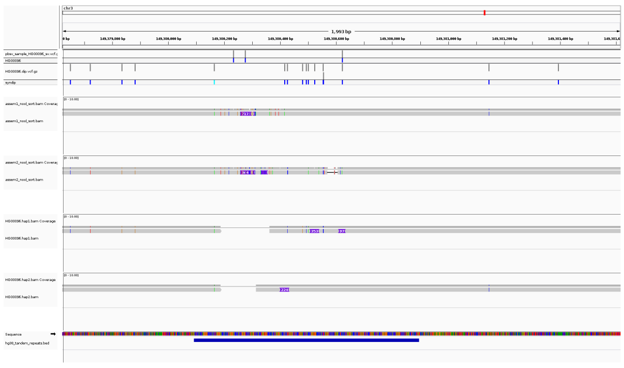

Fig. S64: Category III

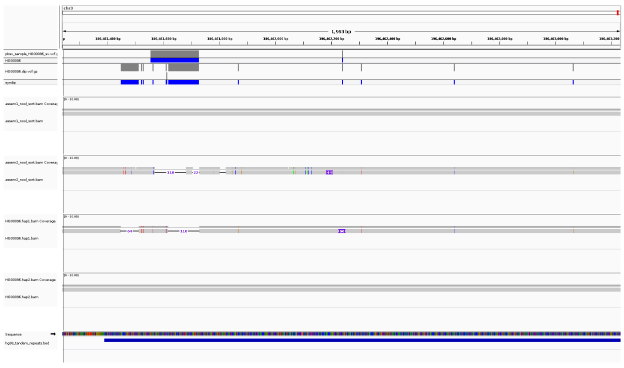

Fig. S65: Category III

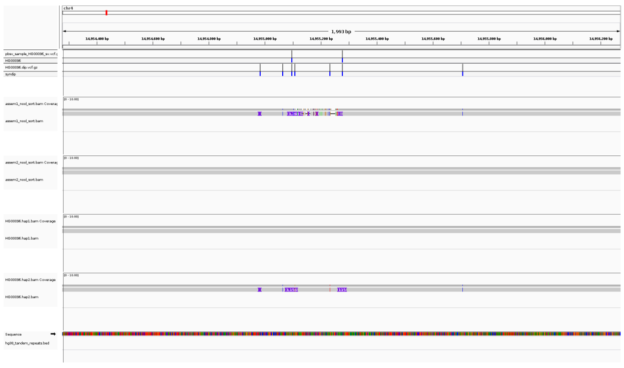

Fig. S66: Category III

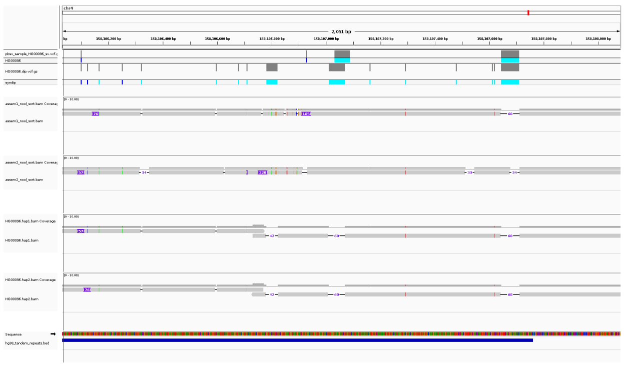

Fig. S67: Category III

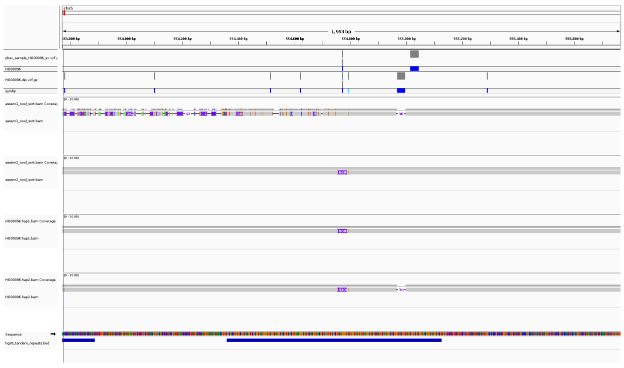

Fig. S68: Category III

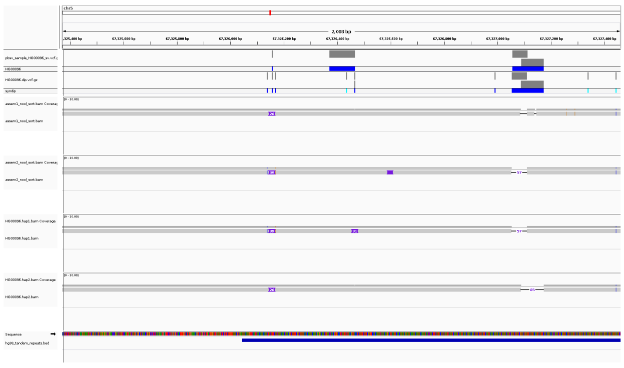

Fig. S69: Category III

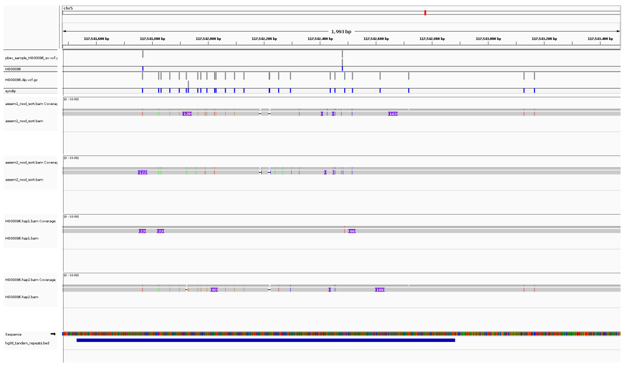

Fig. S70: Category III

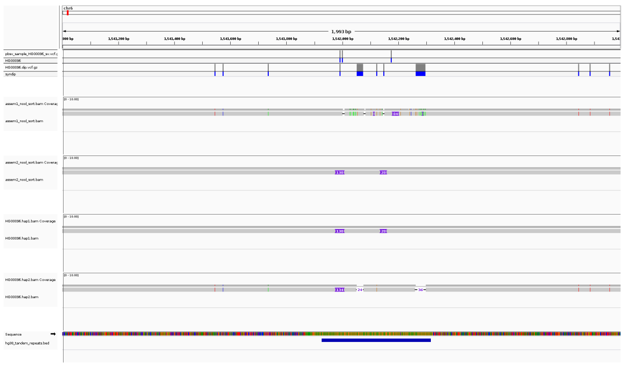

Fig. S71: Category I

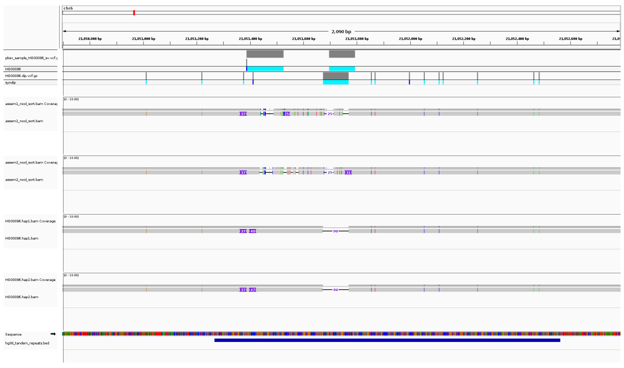

Fig. S72: Category III

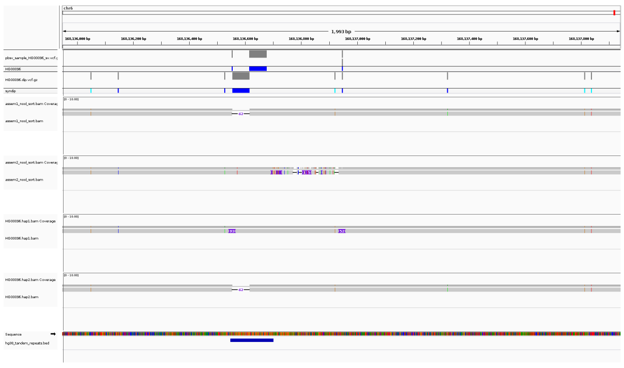

Fig. S73: Category III

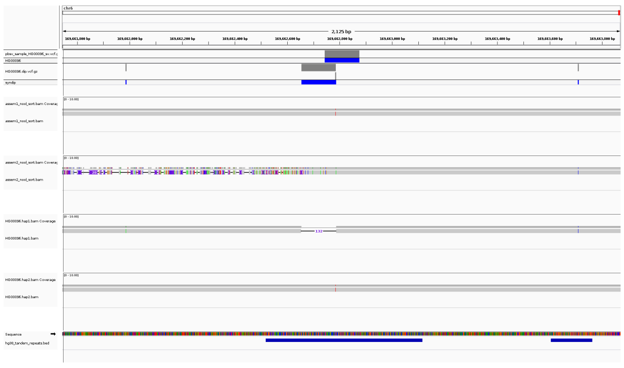

Fig. S74: Category III

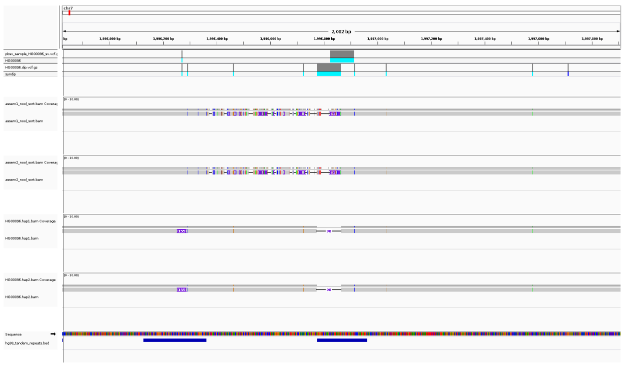

Fig. S75: Category III

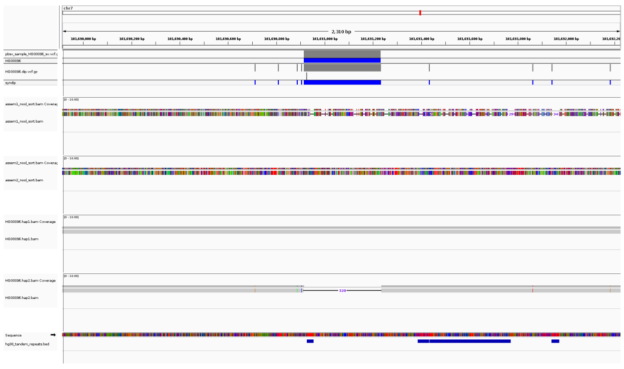

Fig. S76: Category III

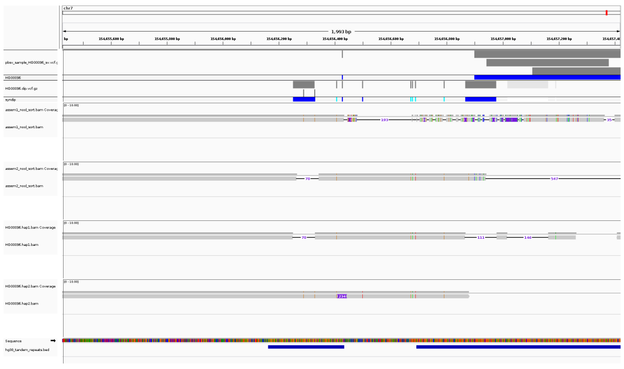

Fig. S77: Category III

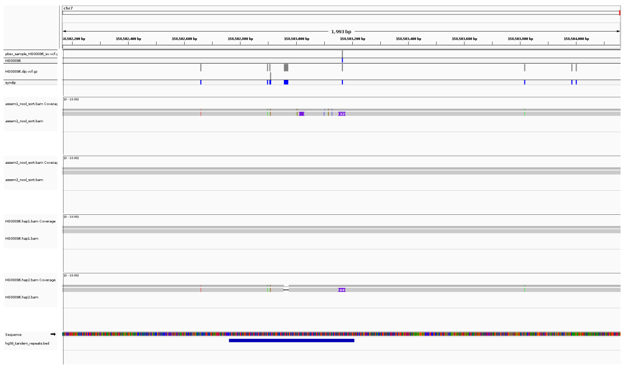

Fig. S78: Category III

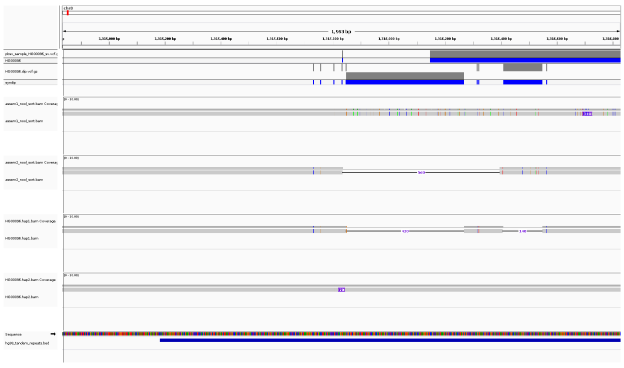

Fig. S79: Category III

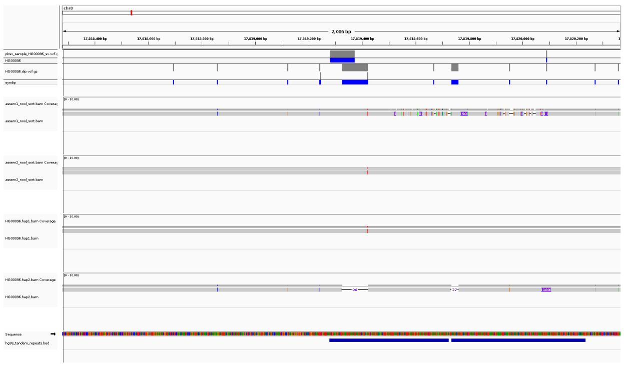

Fig. S80: Category III

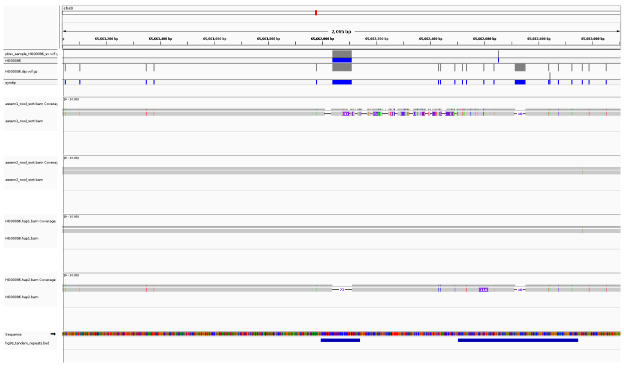

Fig. S81: Category III

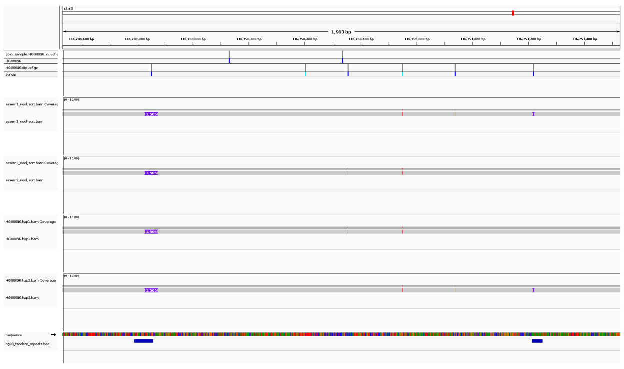

Fig. S82: Category III

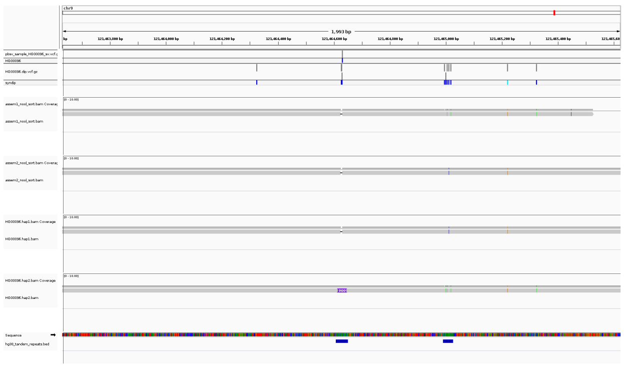

Fig. S83: Category III

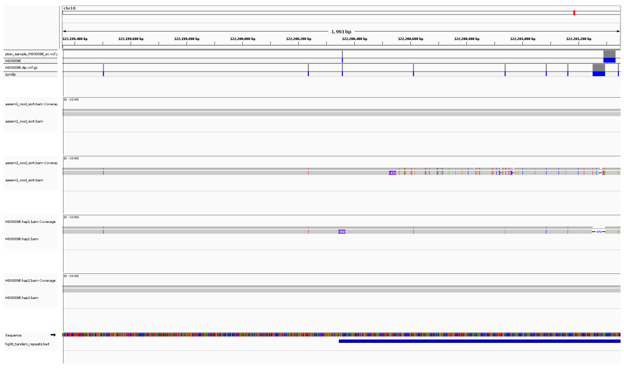

Fig. S84: Category III

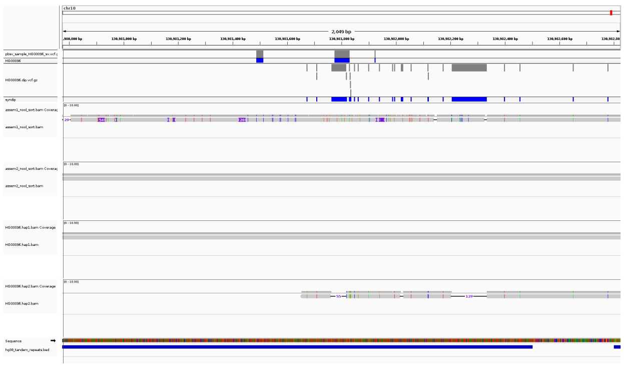

Fig. S85: Category III

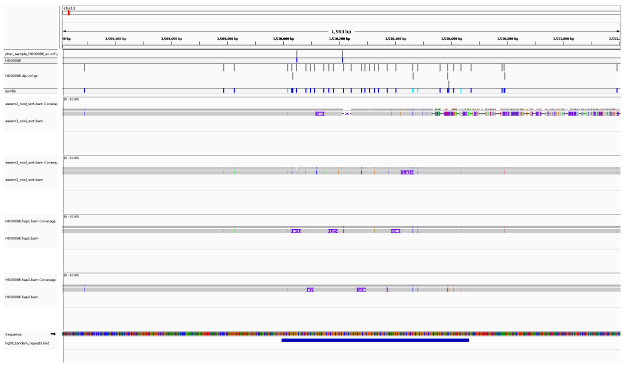

Fig. S86: Category I

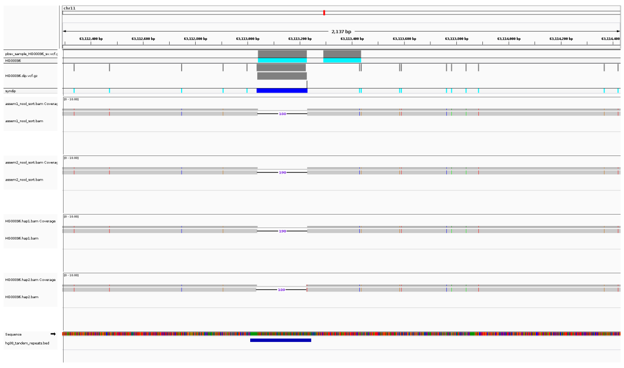

Fig. S87: Category III

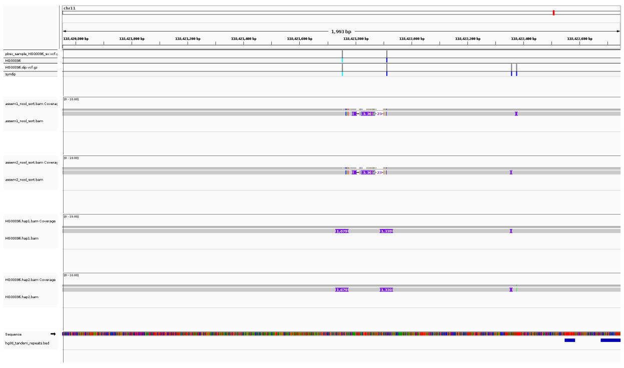

Fig. S88: Category III

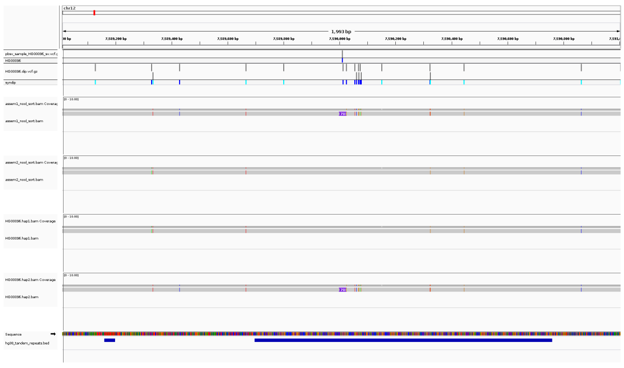

Fig. S89: Category II

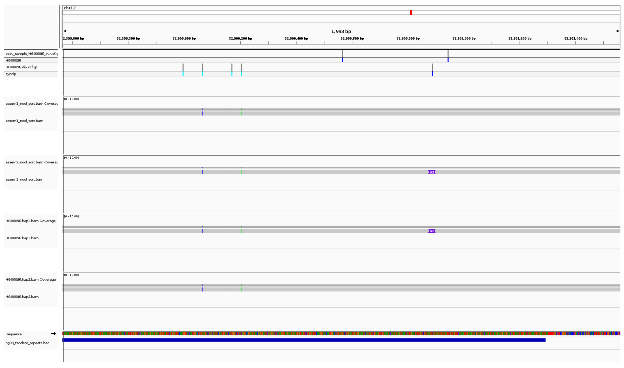

Fig. S90: Category II

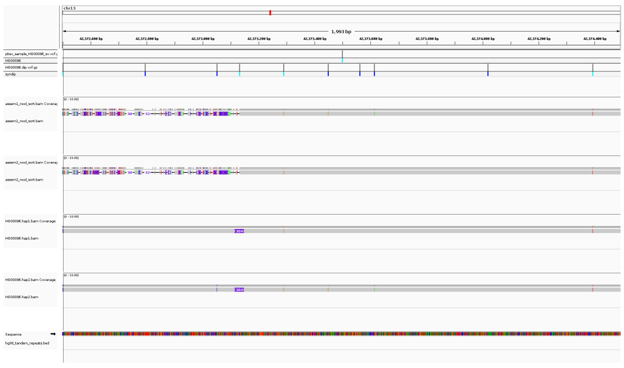

Fig. S91: Category III

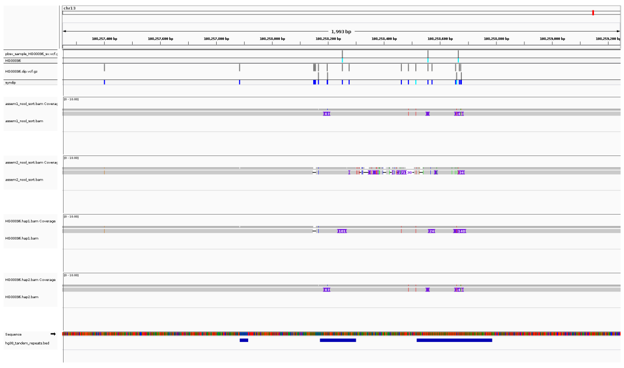

Fig. S92: Category III

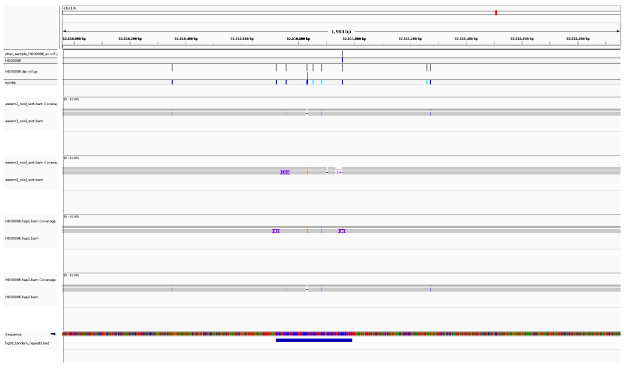

Fig. S93: Category III

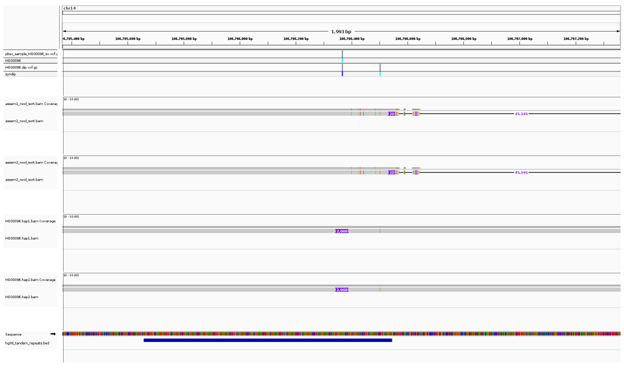

Fig. S94: Category III

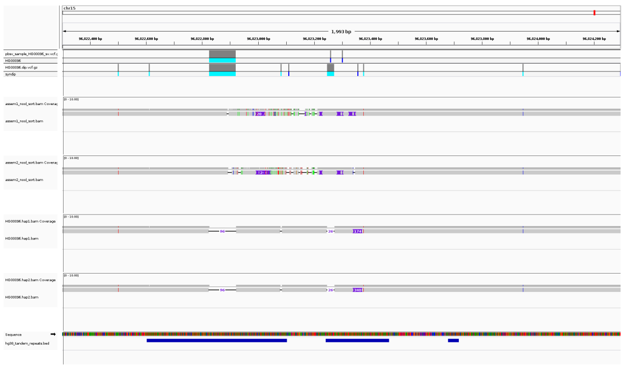

Fig. S95: Category III

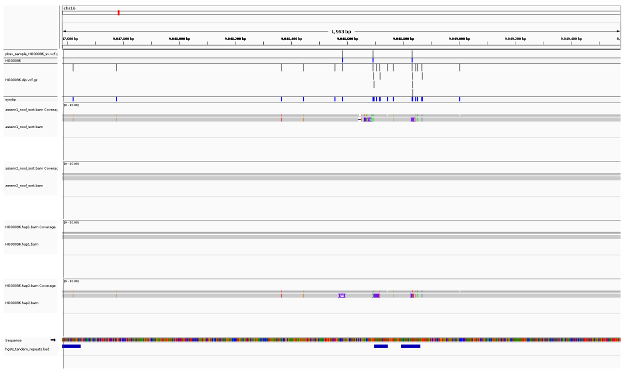

Fig. S96: Category III

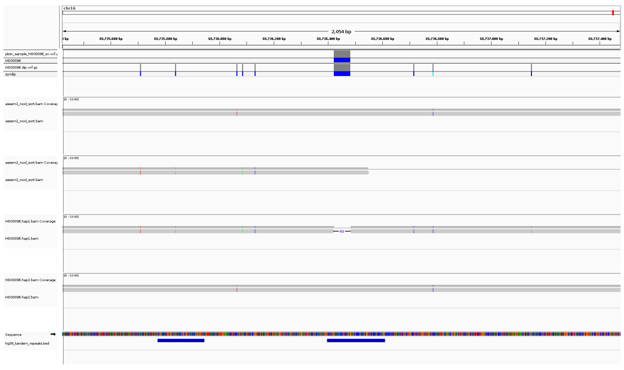

Fig. S97: Category III

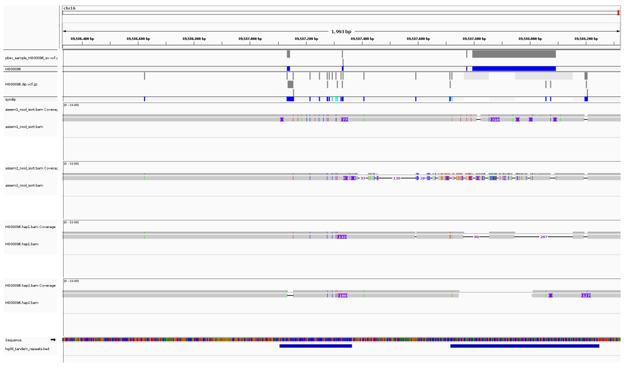

Fig. S98: Category III

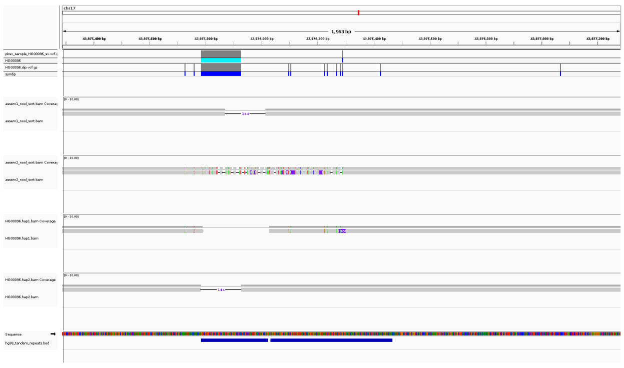

Fig. S99: Category III

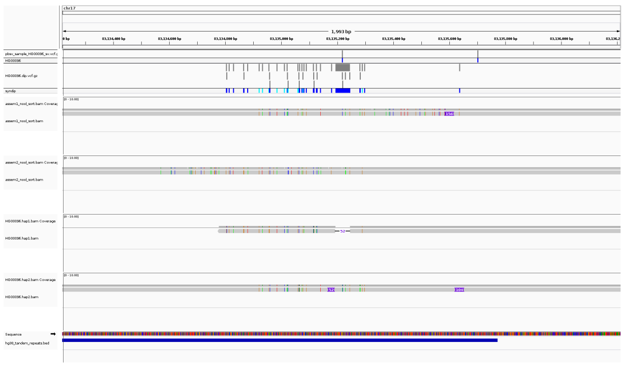

Fig. S100: Category III

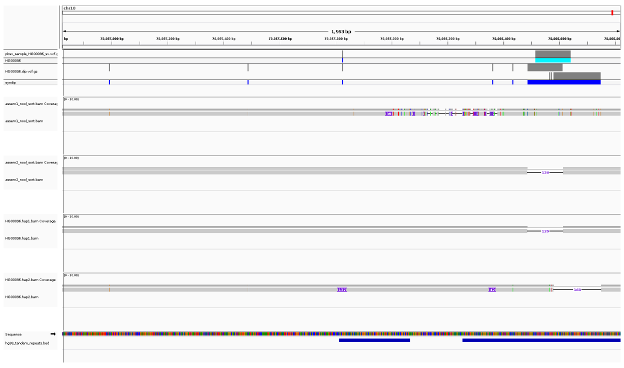

Fig. S101: Category III

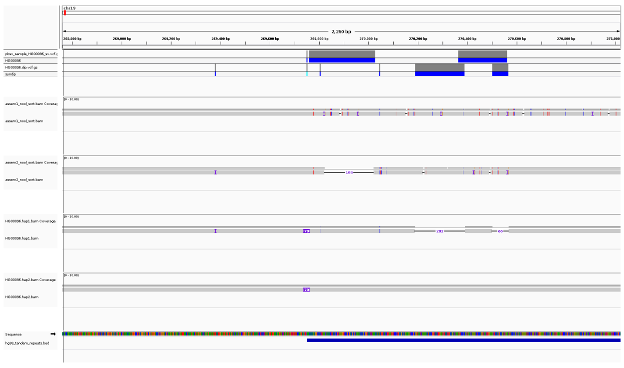

Fig. S102: Category II

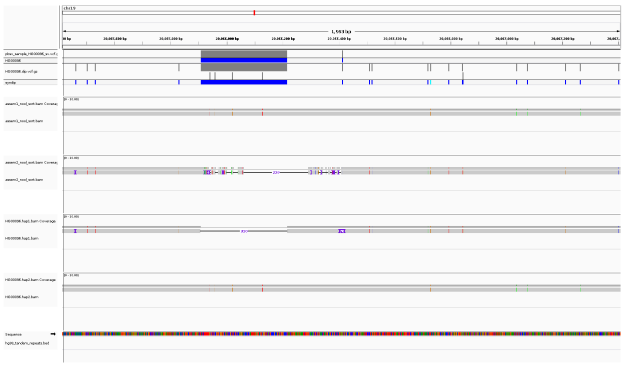

Fig. S103: Category III

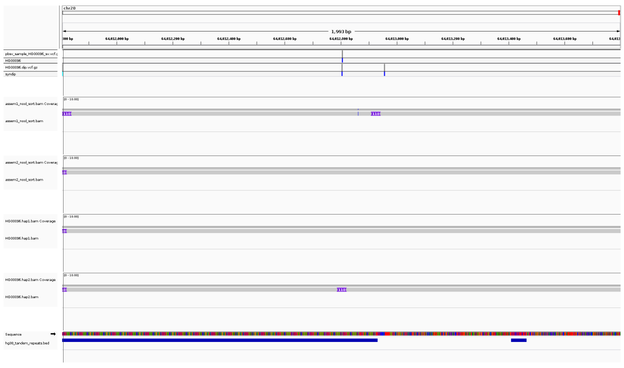

Fig. S104: Category II

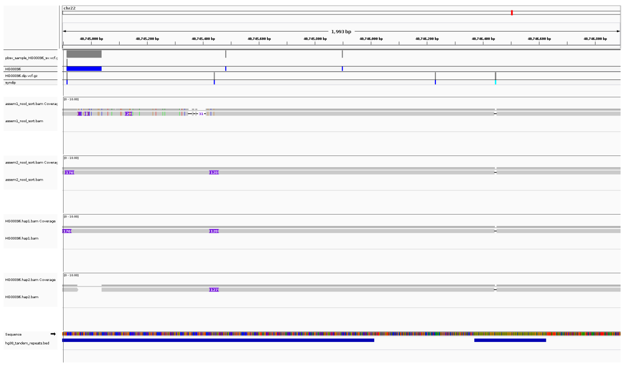

Fig. S105: Category II

6 SV operation

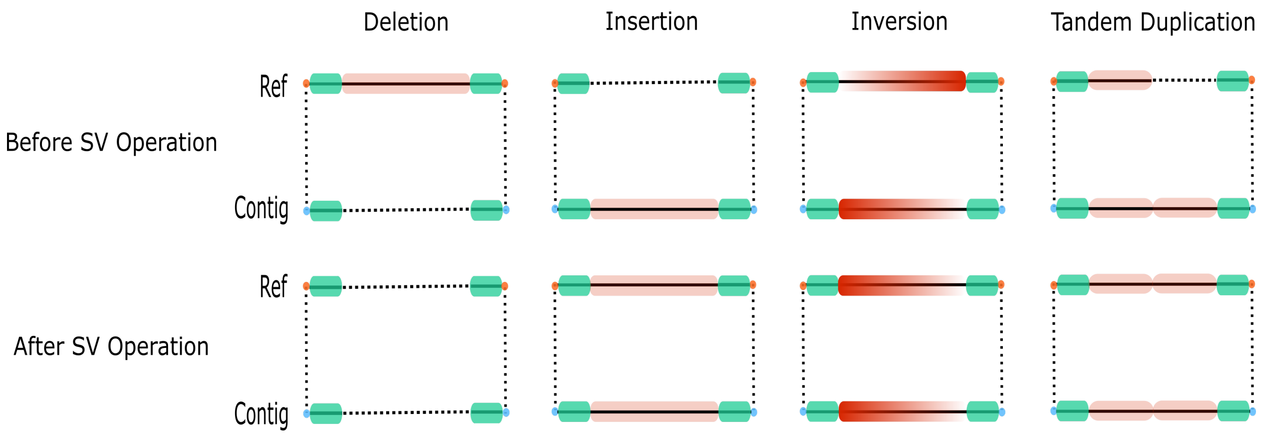

Fig. S106: An illustration of the classes of SV operations considered by TT-Mars, and the result of the reference before and after each operation. Because the reference is not modified to validate an interspersed duplication, it is not shown here.

7 Assembly quality control

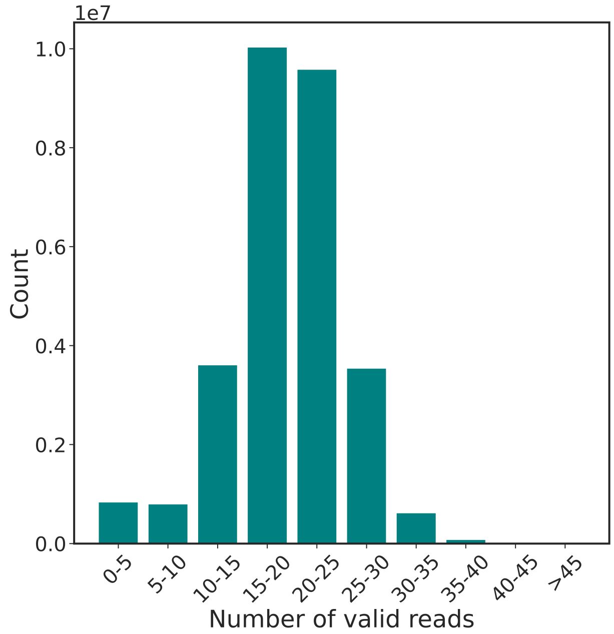

(a) Assembly score haplotype 1

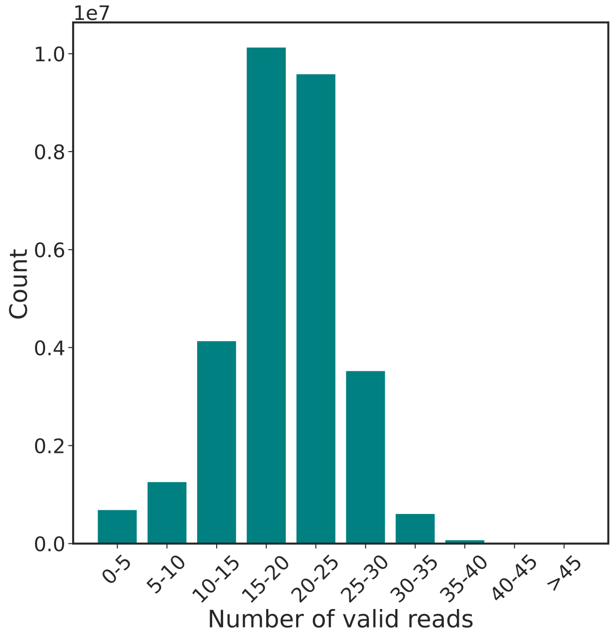

(b) Assembly score haplotype 2

Fig. S107: The distribution of assembly scores across the sample genome HG002, defined by contiguity of sequence alignment. We use Pacbio reads to assess the assembly scores. Raw reads (before haplotype partition) are mapped back to the assemblies using lra with the options `-CCS/CLR` -p s. The genome is divided into 100-base bins. A read that spans a bin with at least 1kb flanking length on both sides is considered as a valid read. We count the number of valid reads as the quality score for each bin. Noisy alignment regions and low-coverage regions are given low quality scores.

8 Command used to run the callers, TT-Mars and truvari

8.1 Callers

Wham: we followed the suggested command.

```
export EXCLUDE=GL000207.1,GL000226.1,GL000229.1,GL000231.1,GL000210.1,\
GL000239.1,GL000235.1,GL000201.1,GL000247.1,GL000245.1,GL000197.1,\
GL000203.1,GL000246.1,GL000249.1,GL000196.1,GL000248.1,GL000244.1,\
GL000238.1,GL000202.1,GL000234.1,GL000232.1,GL000206.1,GL000240.1,\
GL000236.1,GL000241.1,GL000243.1,GL000242.1,GL000230.1,GL000237.1,\
GL000233.1,GL000204.1,GL000198.1,GL000208.1,GL000191.1,GL000227.1,\
GL000228.1,GL000214.1,GL000221.1,GL000209.1,GL000218.1,GL000220.1,\
GL000213.1,GL000211.1,GL000199.1,GL000217.1,GL000216.1,GL000215.1,\
GL000205.1,GL000219.1,GL000224.1,GL000223.1,GL000195.1,GL000212.1,\
GL000222.1,GL000200.1,GL000193.1,GL000194.1,GL000225.1,GL000192.1,NC_007605
whamg -e $EXCLUDE -a ref.fa -f sample.bam | perl utils/filtWhamG.pl > sample.vcf
2> sample.err
```

LUMPY: we followed the suggested command.

```
samtools view -b -F 1294 sample.bam > sample.discordants.unsorted.bam
samtools view -h sample.bam\
| scripts/extractSplitReads_BwaMem -i stdin\
| samtools view -Sb - > sample.splitters.unsorted.bam
samtools sort sample.discordants.unsorted.bam sample.discordants
samtools sort sample.splitters.unsorted.bam sample.splitters
lumpyexpress -B sample.bam -S sample.splitters.bam\
-D sample.discordants.bam -o sample.vcf
```

DELLY: we followed the default command.

```
delly call -x human.hg38.excl.tsv -o sample.bcf -g ref.fa sample.bam
```

pbsv: we followed the default command for sample HG002.

```
pbsv call ref/human_hs37d5.fasta tools/pbsv/*.svsig.gz tools/pbsv/hg2.pbsv.vcf --ccs
bgzip tools/pbsv/hg2.pbsv.vcf
tabix tools/pbsv/hg2.pbsv.vcf.gz
```

dipcall: we followed the default command.

For female samples:

```
dipcall.kit/run-dipcall prefix ref.fa paternal.fa.gz maternal.fa.gz > prefix.mak
make -j2 -f prefix.mak
```

For male samples, PARs on the reference chrY are hard masked:

```
dipcall.kit/run-dipcall -x dipcall.kit/hs38.PAR.bed prefix ref.fa paternal.fa.gz\
maternal.fa.gz > prefix.mak
make -j2 -f prefix.mak
```

8.2 TT-Mars

TT-Mars:

```
sample=sample
reference=path-to-reference_file/hg38.no_alts.fasta
vcf_file=path-to-target-vcf/callset.vcf
asm_h1=path-to-assemblies/h1.fa
asm_h2=path-to-assemblies/h2.fa
output_dir=output-directory
files_dir=./ttmars_files/$sample
centro_file=centromere_hg38.txt
tr_file=hg38_tandem_repeats.bed
num_X_chr=1

python ttmars.py $output_dir $centro_file $files_dir/assem1_non_cov_regions.bed\
$files_dir/assem2_non_cov_regions.bed $vcf_file $reference $asm_h1 $asm_h2\
$files_dir/lo_pos_assem1_result_compressed.bed\
$files_dir/lo_pos_assem2_result_compressed.bed\
$files_dir/lo_pos_assem1_0_result_compressed.bed\
$files_dir/lo_pos_assem2_0_result_compressed.bed $tr_file

python combine.py $output_dir $num_X_chr
```

8.3 Truvari

Truvari:

For short-read callers:

```
truvari bench -f /reference/human_hs37d5.fasta --includebed\
HG002_SVs_Tier1_v0.6.bed -b HG002_SVs_Tier1_v0.6.vcf.gz -c HG002.vcf.gz -o\
./HG002/ --sizemax 10000000 --passonly --pctsim=0 -r 1000 --multimatch &
```

For long-read callers:

```
truvari bench -f /reference/human_hs37d5.fasta --includebed\
HG002_SVs_Tier1_v0.6.bed -b HG002_SVs_Tier1_v0.6.vcf.gz -c HG002.vcf.gz -o\
./HG002/ --sizemax 10000000 --passonly -r 1000 --multimatch &
```
